# Magnetic Properties of Zig-Zag-Edged Hexagonal Nanohelicenes: A Quantum Chemical Study

**DOI:** 10.3390/nano13030415

**Published:** 2023-01-19

**Authors:** Vitaly Porsev, Robert Evarestov

**Affiliations:** Department of Quantum Chemistry, St. Petersburg State University, 199034 Saint Petersburg, Russia

**Keywords:** line symmetry groups, helical symmetry, DFT, helicene, graphene spiral, spontaneous symmetry breaking, metal-insulator transition

## Abstract

The atomic structure and electronic and magnetic properties of two zig-zag-edged hexagonal nanohelicenes of the second type [1.2] and [2.2] were studied by the density functional theory. These objects possess a helical periodicity and belong to the fifth family of line symmetry groups in their global energy minimum. These nanohelicenes were shown by us to be diamagnetic metals that undergo spontaneous symmetry breaking into antiferromagnetic semiconductors as a result of the Mott–Hubbard metal-insulator transition. However, under some torsional stress, a reversible transformation to a diamagnetic metal can take place, which is promising for the use of nanohelicenes in electro-magneto-mechanical nanodevices.

## 1. Introduction

Helicity is a phenomenon widespread in nature. In particular, the basic elements of living organisms, DNA and RNA, have a helical topology. In recent years, a variety of methods have been developed that make it possible to synthesize helical nanoobjects [1]. Among them, we can mention chiral nanorods of various structures [2], chiral nanotubes, in particular, carbon nanotubes [3], and nanotube nanoropes [4]. The polymer chemistry of helically ordered polymers [5] is also rapidly developing: polyacetylenes [6], polythiophenes [7], polyfurans [8], and polytrypticenes [9] are just a small number of examples of such systems. Special mention should be made of polymer systems with a continuous conjugated π-system, in particular, nanohelicenes and carbon nanoribbons [10]. Nanohelicenes, NHs, (the names graphene spirals and graphene helicoids are also used in the literature) are systems that extend the idea of helicene twisting [11] to infinity. For the first time, polymer systems with an NH skeleton were obtained quite recently [12,13,14]. Using the basic skeleton, many variations of NHs can be modeled, for example, by the expansion of the *shaft* and the extension of the *ribbon* [15].

The properties of NHs and their derivatives are actively studied by theoretical methods due to the unique natural spring topology, which leads to great opportunities in the reversible mechanical modulation of various properties of NHs. Among such works, we note the study of the mechanical properties of NHs by the methods of molecular dynamics [16,17,18,19,20,21,22,23,24,25,26,27,28]. The electronic structure of NHs of various types was studied by semi-empirical DFTB methods [29,30,31] and non-empirical DFT methods [32,33,34,35,36,37,38,39,40,41,42]. It has been shown that various families of NHs are possible, including diamagnetic semiconductors, antiferromagnetic semiconductors, ferromagnets, and metals. This diversity, combined with the spring structure and helically coiled continuous π-system, makes NHs very promising for future applications in nanotechnology.

Let us briefly consider the nomenclature of zig-zag-terminated hexagonal [*m*.*n*] helicenes (for more details, see [38]). The *width of the ribbon* in hexagons is given by the index *n* and the *radius of the shaft* in hexagons by the index *m*. The parity of *n* determines the type of NH. If *n* is odd, then the NH is a diamagnetic semiconductor; NHs with an even *n* are metals that are unstable against the transition to an antiferromagnetic semiconductor [38]. The *m* index changes only the quantitative characteristics of NHs. For example, an increase in m leads to a decrease in the electronic band gap, *E*_gap_ [38].

It should be noted that all the theoretical studies mentioned above (including [38]) did not take into account the torsional degree of freedom, which is characteristic of such systems. All of the theoretical studies were carried out under the assumption that each coil of the NH is located exactly above the previous one. However, it is known from the data of X-ray diffraction analysis of molecular helicenes [43] that this assumption is incorrect; the coils are additionally twisted relative to each other, and the direction of the twisting, as well as its magnitude, is a priori unknown and requires an additional study.

In our works [44,45], an algorithm was proposed for calculating the twisting properties of such structures by ab initio methods based on the theory of line symmetry groups [46,47,48,49,50,51]. The algorithm was tested on a group of NHs with an odd *n* [44,45]. In addition, the algorithm was applied to the quantum chemical study of other helically periodic structures, such as polytwistanes [52] and helical polyacetylenes [53]. All mentioned nanostructures belong by symmetry to the first or fifth family of the line symmetry groups, which means that in fact, they are examples of helically periodic structures without translational periodicity [54]. Due to the presence of planes of symmetry, the line groups of the remaining 11 families always include translational symmetry [50]. The presence of symmetry planes leads to the rationality of the twist angle, and their absence makes possible irrational values of the angle and the absence of translational periodicity (see details in Section 2.1).

**Figure 1:**
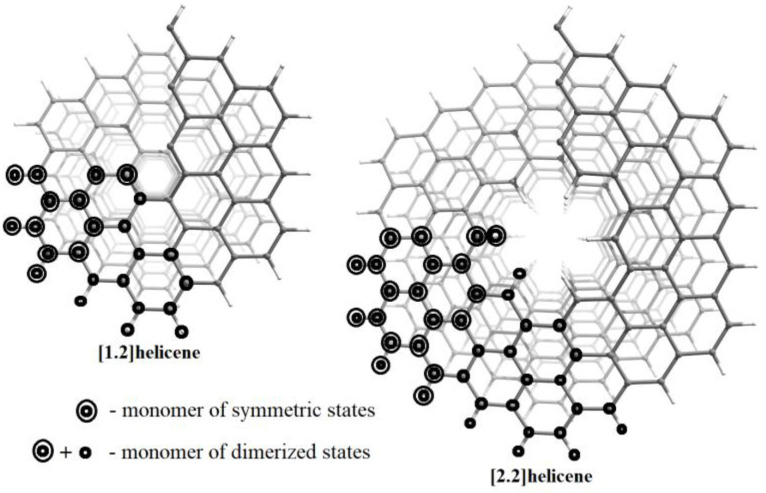
Top view of the atomic structure of NHs under consideration. The symmetry groups of the presented structures are *L*6_1_ or *L*3_1_ for symmetric or dimerized states, respectively. White and gray colors represent hydrogen and carbon atoms, respectively.

Depending on the edge structure, the hexagonal NHs are classified as zig-zag- or arm-chair-terminated ones. Here, the results of a theoretical study of the atomic, electronic, and magnetic structure of zig-zag-terminated hexagonal NHs with an even *n* are presented: two NHs with *n* = 2 and *m* = 1,2 were under consideration (see Figure 1). The most symmetric state of NHs with an even *n* has metallic properties, so it is necessary to take into account the possibility of spontaneous symmetry breaking, which is realized through the metal-insulator transition (MIT). Two types of MIT are possible for metallic quasi-one-dimensional (Q1D) nanosystems—the Peierls MIT (electron–phonon interactions [55]) and the Mott–Hubbard MIT (electron–electron interactions [56]). It was shown that the transition of the Mott–Hubbard type is most advantageous [38]. It was also shown in [41] that the Mott–Hubbard type transition is beneficial for the carbon skeleton of nanohelicene (i.e., without saturation of free carbon valences by hydrogen atoms), indirectly confirming the correctness of the results of [38].

We should note our results of [53], in which helical polyacetylenes were studied. It was shown that helical polyacetylenes could be formally considered as NHs with an *n* = 0. It is known that the highly symmetrical state of polyacetylene is a metal, which means that spontaneous symmetry breaking is expected. This result has been obtained by us for helical polyacetylenes.

## 2. Computational Details

### 2.1. Line Symmetry Groups

In the line symmetry group, ***L***, theory [50] a *roto-helical factorization* is used:

(1) L=ZP

The point symmetry of a monomer compatible with helical symmetry is described by the group ***P***. Thirteen families of line groups are possible [50]. In the case of the first family of line symmetry groups, only pure rotations about the helical axis are operations of ***P***. Here, only NHs with one helix are considered, so ***P*** = ***C***_1_, and the line symmetry group of the NHs coincides with the helical factor, ***L*** = ***Z***. If the NHs are invariant under rotations by an angle of 180° around the *U* axes, perpendicular to the principal axis, Oz, the point group is ***P*** = ***D***_1_, and the line symmetry group is ***L*** = ***Z*** ∧ ***D***_1_ (the fifth family). It should be noted that only the first or fifth families are compatible with the incommensurate helical factor ***Z***.

*Z* is called a *generalized* translation and is written as *Z* = (*X*|*f*), where *X* is either the rotation, *C_Q_*, or the reflection, *σ_V_*, and *f >* 0 is the *partial translation* along the *z*-axis. Elements of *Z* act on the *monomer*, mapping it onto the adjacent one and thus generating an infinite cyclic group, ***Z***. In the case of NHs, *X* can be only *C_Q_*, and the group ***Z*** is called helical. *C_Q_* is an operation of rotation around the *z*-axis by a *rotation angle* determined by *Q* (the order of the helical axis):

(2) $$\varphi =\frac{360{}^{\circ}}{Q},\;Q\geq 1$$

The translational periodicity appears only when *Q* is rational, i.e.,

(3) $$Q=\frac{q}{r},\mathrm{where}\;q>r\;\mathrm{and}\;q,r—\mathrm{coprime}.$$

Then, the rotation angle in such cases is equal to:

(4) $$\varphi =\frac{r\cdot 360{}^{\circ}}{q}$$

When *Q* is rational, then, in addition to the helical periodicity (the rotations around the screw axis of order *q*), there is a translational periodicity with a translational period, *T*:

(5) T=qf.

In this case, ***L*** = ***TP*** also (the crystallographic factorization), and the L group is called *commensurate* [50].

When *Q* is irrational, the system is called *incommensurate* [50] and has no translational periodicity (but the helical periodicity remains).

### 2.2. Modelling of the Metal-Insulator Transition

The conducting state of Q1D objects is unstable and undergoes a spontaneous symmetry breaking with an electronic band gap arising, i.e., the MIT. Two types of MIT are known. In the case of the Peierls MIT [55], a *bond order wave* arises, i.e., an alternation of the single and double bonds from the initially equivalent *aromatic* C-C bonds of the conducting state. The neighboring atoms of the same orbit (i.e., the set of atoms linked together by symmetry group operations) of the conducting state are no longer equivalent. The decrease in symmetry occurs due to the decrease in the symmetry of the nuclear core. In the case of the Mott–Hubbard MIT [56], the nuclear core does not change the initial symmetry, the electronic band gap appears, and the energy decreases due to the spin degrees of freedom. An alternating spin density occurs (the *spin density wave*) on two adjacent monomers that are no longer equivalent.

The study of the antiferromagnetic states requires the application of the magnetic line groups theory [57]. In the case of the first family of line symmetry groups with ***P*** = ***C***_1_, only *klassengleiche* magnetic groups are possible:

(6) $$L_{mag}=Z^{\prime }+\theta Z^{\prime }.$$

Here, *θ* is the time reversal operation, and ***Z’*** is a subgroup of ***Z*** of index 2. Group ***Z*** is a line symmetry group of an initial metallic structure.

Thus, the simulation of both types of MIT can be performed in a similar way. If *Q* and *f* are parameters of the initial structure, then the dimerization of the neighboring monomers (the Peierls MIT) or the possibility of an alternating spin on the neighboring monomers (the Mott–Hubbard MIT) leads to a doubling of *f* and a halving of the helical axis order *Q*:

(7) $$Q^{\prime }=\frac{Q}{2}\;\mathrm{and}\;f^{\prime }=2f.$$

There are no physical reasons to consider *Q* and *Q*′ as rational. Nevertheless, the method used [44] is based on an interpolation between the rational orders of the helical axis, so *Q* and *Q*′ should be rational. Since a rational number can be represented as a ratio of two coprime integers, then $Q=\frac{q}{r}$ (Equation (3)), and *Q*′ must be equal to $Q^{\prime }=\frac{q}{2r}$. There are only two variants:

1. *q* is even, $q^{\prime }=\frac{q}{2}$, $Q^{\prime }=\frac{q^{\prime }}{r}$. Parameters *q*’ and *r* are coprime integers.
2. *q* is odd, $r^{\prime }=2r$, $Q^{\prime }=\frac{q}{r^{\prime }}$. Parameters *q* and *r*’ are coprime integers.

If C*_x_*H*_y_* is the formula of the monomer in the initial structure, then the monomer of the dimerized states (the dimer) is C_2*x*_H_2*y*_. The atoms of the dimer are atoms of the initial monomer and the next neighboring monomer (see Figure 1). When the dimer calculations are performed by the DFT (Kohn–Sham) method without spin polarization, the results obtained are diamagnetic and correspond to the Peierls MIT. Using the spin-polarized DFT, one can obtain the antiferromagnetic ordering of the dimerized state, which describes the Mott–Hubbard MIT. It should be noted that our calculations of the electron density are self-consistent (the Kohn–Sham scheme). This means that if the diamagnetic state is lower in energy than the antiferromagnetic state, then a diamagnetic state will be obtained as a result of self-consistency. The Mott–Hubbard and Peierls MITs are qualitative models that make it possible to understand the physical meaning of the ab initio DFT results. The presented scheme of the modeling of the MIT was previously used by us in the case of helical polyacetylenes [53].

### 2.3. Calculation Parameters

The CRYSTAL17 software package was used for the calculations [58,59]. This suite is designed for high-quality ab initio calculations of the atomic and electronic structure and other properties of periodic systems of various dimensions, in particular, systems with helical symmetry [60]. The hybrid PBE0 functional was used [61]. The pob-TZVP atomic basis set (a full electron) was used [62]. The total electronic energy per cell was calculated with a convergence threshold of 10^−9^ Ha. The truncation criteria 10^−7^, 10^−7^, 10^−7^, 10^−7^, and 10^−14^ were used for two-electron integrals of the Hartree–Fock exchange and Coulomb series (when the overlap between two atomic orbitals is smaller than the criterion given, the corresponding integral is disregarded or evaluated in a less precise way). Both the translational period and atomic positions have been optimized. The gradient threshold was set equal to 0.0003 Ha∙Bohr^−1^. The Monkhorst–Pack [63] integration method of the one-dimensional Brillouin Zone was used with a 32 **k**-point sampling.

## 3. Results and Discussion

### 3.1. Energy Minima and Atomic Structure

The coexistence of several states makes the quantum chemical study of NHs [*m*.*n*] of the second type (with an even *n*) much more complicated than the earlier calculations of NHs of the first type (with an odd *n*), which are diamagnetic semiconductors [44].

For each NH under study, it is necessary to consider the monomer state, which is a diamagnetic metal, “mono” [38]. A spin-polarized calculation of this symmetric state can lead to the appearance of a ferromagnetic ordering, i.e., the “ferro” state.

The study of the MIT requires the calculation of a system with a doubled monomer (the dimer). The dimer calculation without spin polarization allows one to investigate the Peierls MIT. Such a state we call a “dim” state. The dimer calculation with spin polarization allows the manifestation of the Mott–Hubbard MIT with the appearance of an antiferromagnetic state, “af”.

It should be noted that spin-polarized calculations may be made using a guess, in which the expected version of the spin ordering is specified in the input for the self-consistent calculation. In particular, such a possibility exists in the CRYSTAL17 code [58,59] used by us for NHs’ calculations (see below). It is important to emphasize that the final electronic structure is self-consistent; no additional spin-restriction conditions are imposed during the self-consistent calculation. Therefore, the final electronic structure calculated after a preliminary guess of the spin distribution can be either spin-polarized or diamagnetic, whichever state is more favorable by the energy. In the latter case, the result obtained will coincide with the results of the corresponding calculation without spin polarization. Thus, if spin polarization is unfavorable, then the calculation with the preliminary guess “ferro” or “af” will converge to the electronic structure of the “mono” or “dim” states, respectively.

Thus, for [1.2] helicene and [2.2] helicene, the structure and the properties were calculated in the four different states mentioned. We used the monomers C_9_H_3_ and C_15_H_5_ and the dimers C_18_H_6_ and C_30_H_10_ for the [1.2] helicene and [2.2] helicene, respectively.

To receive the energy dependence on the angle of the helical rotation for the irrational *Q* (infinite NHs), we made calculations for the translationally periodic monomers and dimers (rational *Q* values).

Table 1 gives for [1.2] and [2.2] NHs the values of the rational *Q* for the different choices of the *q*, *p*, *r*, and rotation angle, φ, (see Equations (2)–(5)). The choice of the q and r values was determined by two main factors: (i) the resulting *Q* should be close to the desired values, and (ii) the *q* parameter, which determines the number of monomers in the periodic cell, should be minimal in order to reduce the computation time. The parameter *p*-values are found for given *q*, *r* values using the relation:

(8) rp−1=ql,wherelissomepositiveinteger.

Equation (8) connects crystallographic and helical factorizations of the line groups [50]. The calculations were made for translationally periodic NHs with the line group symmetry *L*q_p_.

The use of the data in Table 1 allows one to consider *Q* (and the corresponding φ) in the intervals:

- [1.2]NH_mono_; $Q\in \left[5\frac{1}{5},\;6\frac{2}{3}\right]$ and φ∈[54°, 69.231°] with monomer C_9_H_3_;
- [1.2]NH_ferro_; $Q\in \left[5\frac{1}{5},\;6\frac{2}{3}\right]$ and φ∈[54°, 69.231°] with monomer C_9_H_3_;
- [1.2]NH_dim_; $Q\in \left[2\frac{3}{5},\;3\frac{1}{3}\right]$ and φ∈[108°, 138.462°] with monomer C_18_H_6_;
- [1.2]NH_af_; $Q\in \left[2\frac{3}{5},\;3\frac{1}{3}\right]$ and φ∈[108°, 138.462°] with monomer C_18_H_6_;
- [2.2]NH_mono_; $Q\in \left[5\frac{1}{2},\;6\frac{2}{3}\right]$ and φ∈[54°, 65.455°] with monomer C_15_H_5_;
- [2.2]NH_ferro_; $Q\in \left[5\frac{1}{2},\;6\frac{2}{3}\right]$ and φ∈[54°, 65.455°] with monomer C_15_H_5_;
- [2.2]NH_dim_; $Q\in \left[2\frac{3}{4},\;3\frac{1}{3}\right]$ and φ∈[108°, 130.909°] with monomer C_30_H_10_;
- [2.2]NH_af_; $Q\in \left[2\frac{3}{4},\;3\frac{1}{3}\right]$ and φ∈[108°, 130.909°] with monomer C_30_H_10_.

To obtain the torsion curves of the relative energy per monomer, *E_rel_*(*state*, φ), the energies of [1.2]NH_af_ and [2.2]NH_af_ with *Q* = 3 were chosen as zero values (i.e., NHs with an *L*3_1_ symmetry). Figure 1 shows a top view of the structure of these NHs. Then, the *E_rel_*(*state*, φ) is:

(9) $$E_{rel}\left(state,\varphi \right)=\frac{E\left(af,\;120{}^{\circ}\right)}{3}-k\frac{E_{tot}\left(state,\varphi \right)}{q}$$

Here, the $E_{tot}\left(state,\varphi \right)$ is the total energy of the “state” per elementary cell (the translationally periodic) at an angle φ, q—the parameter from (Equation (2)), showing the number of monomers of the “state” per elementary cell at φ. Parameter *k* = 1 for “af” and “dim”, and *k* = 2 for “mono” and “ferro”.

**Figure 2:**
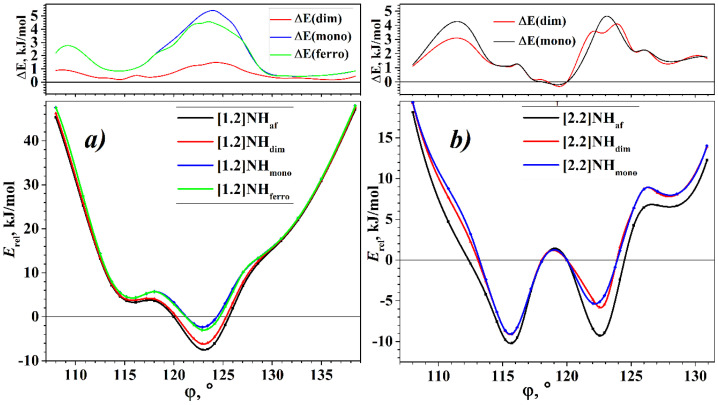
Torsion energy curves of NHs, *E_rel_*(*state*, φ), and torsion curves of energy differences between states, Δ*E*(*state*, φ ) = *E_rel_*(*state*, φ )–*E_rel_*(*af*, φ ). (**a**) [1.2]helicene; (**b**) [2.2]helicene.

The torsion energy curves of different states are shown in Figure 2. Let us first consider the torsion dependence of the energy of the [1.2]NH states. In the entire range, the “mono” state has the highest energy, which shows the instability of the metallic state with respect to spontaneous symmetry breaking. The “af” state has the lowest energy over the entire range studied, indicating that the [1.2]helicene is a pure Mott–Hubbard semiconductor. However, the Peierls MIT also is possible since the “dim” state is lower in energy than the “mono” in the entire considered interval. It is important to note that for [1.2]helicene the “dim” state is always higher in energy than “af” state. The situation when the optimization of the “af” state leads to a diamagnetic “dim” is not realized.

However, in the case of the “ferro” state, the appearance of the spin polarization is the result of the self-consistency of the electronic structure only in a small region around the energy minimum, where the energy of the “ferro” state is less than the energy of the diamagnetic “mono” state. Beyond this region, the electronic structure with the initial ferromagnetic ordering leads to a diamagnetic solution of the Kohn–Sham equations, i.e., to the “mono” state.

The general view of the *E_rel_*(φ) curves of the [1.2]helicene states is similar to the *E_rel_*(φ) curve of the [1.1]helicene (see [44]). This is expected since the largest contribution to the energy for NHs with an *m* = 1 comes from steric hindrances, which are most pronounced for the internal atoms of the NH’s ribbon. Accordingly, for all four states of the [1.2]helicene, the minimum energy is localized in the region of φ a little over 60° for the monomer and 120° for the dimer. In other words, the structure of the minimum energy is *overtwisted* relative to the structure with 60° or 120°, in which each coil is located exactly above the previous one. Note that *overtwisting* was previously obtained for the [1.1]helicene, and this effect was confirmed by the experimental data for molecular helicenes [43].

The difference between the energies of the states is maximal in the region of the energy minimum. In this region, the energy gains at the Mott–Hubbard and Peierls MITs relative to the “mono” state are about 5 and 4 kJ/mol, respectively. In other regions, these values are much smaller (see Figure 2). Interestingly, in the case of helical polyacetylenes, the dependence is inverse: in the energy minimum region, the difference between the “mono”, “dim”, and “af” states is minimal (see [53]).

The torsion energy curves of the states of the [2.2]helicene have a more complex structure. First of all, we note that the “ferro” state does not exist for this helicene. In other words, in the entire range of the φ change, the electronic structure with the initial ferromagnetic ordering converges to the diamagnetic “mono” state. For this reason, Figure 2b shows torsion energy curves for three states of the [2.2]helicene: the “mono”, “dim”, and “af” states.

The “af” state of the [2.2]helicene has the lowest energy in the entire φ change region studied, except for a small range of φ values close to 120°. Thus, the [2.2]helicene is a Mott–Hubbard semiconductor, like [1.2]helicene. The energy of the Peierls “dim” state is close to the energy of the symmetric “mono” state and coincides with it at many points of the torsion curve.

Let us take a closer look at the region around φ = 120°. Among the rest of the studied interval, this region is notable due to the fact that at φ = 120° the atoms of one coil are arranged exactly above the corresponding atoms of the neighbor coil. At this point, the calculation of all three states of the [2.2]helicene (i.e., “mono”, “dim”, and “af”) leads to a metallic diamagnetic state, “mono”. A similar result was obtained earlier [38]. Moreover, a similar state degeneracy at φ = 120° is also observed for more complicated NHs of the second type, and it is absent only for the [1.2]helicene. It can be assumed that this degeneracy is a consequence of the *intercoil coupling* since the removal of degeneracy by the transition to an antiferromagnetic semiconductor (the Mott–Hubbard MIT) was shown in [38] at sufficient stretching of NHs of the second type. In [29], the importance of taking into account the interaction between the coils of NHs for obtaining correct results in the calculation by the DFTB method was shown, and in [35,37], the phase transition to the ferromagnetic state of NHs of various shapes under tension was shown also.

The intercoil coupling of the [2.2]helicene, which is characteristic of the region around φ = 120°, can be destroyed not only by tension but also by torsion. According to Figure 2b, both the decreasing and increasing of φ result in the Mott–Hubbard MIT and the appearance of the minima on the torsion energy curve.

There are two minima on the torsion energy curve of the most favorable “af” state of the [2.2]helicene (see Figure 2b). The global minimum corresponds to a structure that is *undertwisted* with respect to φ = 120°, but the local minimum for an *overtwisted* structure has very close energy. It even can be assumed that for the “af” state of the [2.2]helicene, the undertwisted and overtwisted structures are equally probable. Note that for the “mono” and “dim” states, the energy of the undertwisted structure is approximately 3 kJ/mol lower than the energy of the overtwisted structure.

**Figure 3:**
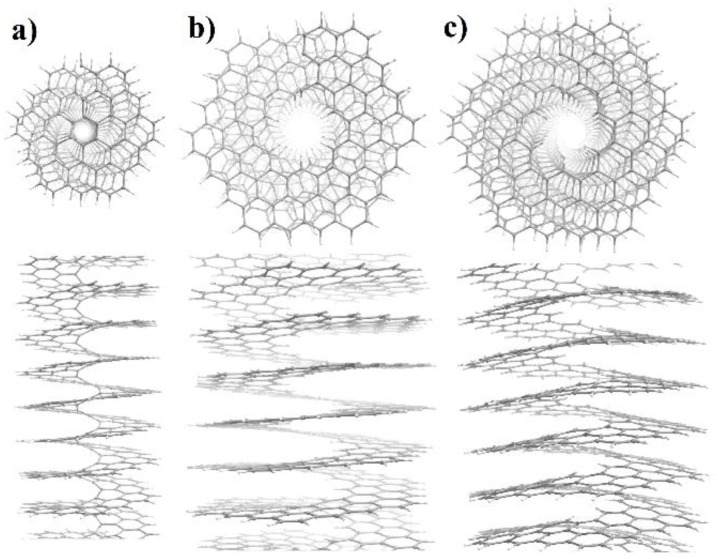
Top and side views of the atomic structures of NHs corresponding to the energy minima. (**a**) [1.2] NH_af_; *E_rel_*(123.086°) = −7.523 kJ/mol. (**b**) [2.2]NH_af_, global minima; *E_rel_*(115.617°) = −10.225 kJ/mol. (**c**) [2.2]NH_af_, local minima; *E_rel_*(122.579°) = −9.284 kJ/mol.

The torsion energy curves shown in Figure 2 were calculated assuming the presence of only a helical axis of symmetry, i.e., that NHs belong to the first family of the line symmetry groups. The optimized atomic structures at the energy minimum of the [1.2] helicene and the two energy minima of the [2.2] helicene are shown in Figure 3. It can be easily seen that for the atomic structure of the [1.2] helicene (Figure 3a) and the atomic structure at the global energy minimum of the [2.2] helicene (Figure 3b), the plane of the ribbon is perpendicular to the helical axis. This leads to the appearance of additional symmetry elements—the rotation axes of the second order, *U*, directed perpendicular to the helical axis. This means that both considered NHs are nanostructures with a symmetry belonging to the fifth family of the line symmetry groups. A similar result was obtained for NHs of the first type (n = 1) (see [44]). Interesting that the atomic structure of the second minimum of the [2.2] helicene (Figure 3c) is characterized by the bending of the ribbon, forming a cone-like structure wrapped around the helical axis. This behavior is obviously associated with the steric hindrances that arise at the outer edge of the ribbon when the rotation angle increases. Such bending appears already at φ = 120° and manifests itself to an increasing extent with a further increase of φ. Thus, under torsion, the symmetry of the NH decreases to the first family of the line symmetry groups.

We also note that this symmetry breaking is not spontaneous, like the MIT, but is induced by an external torsion action. In the case of NHs, spontaneous and torsion-induced symmetry breakings act on various symmetry elements. Spontaneous symmetry breaking halves the order of the helical axis, but the symmetry of NHs continues to belong to the 5th family of the line groups. Torsion-induced symmetry breaking lowers the point symmetry of the monomer from ***D***_1_ to ***C***_1_ while the order of the helical axis is preserved.

### 3.2. Electronic and Magnetic Properties

**Figure 4:**
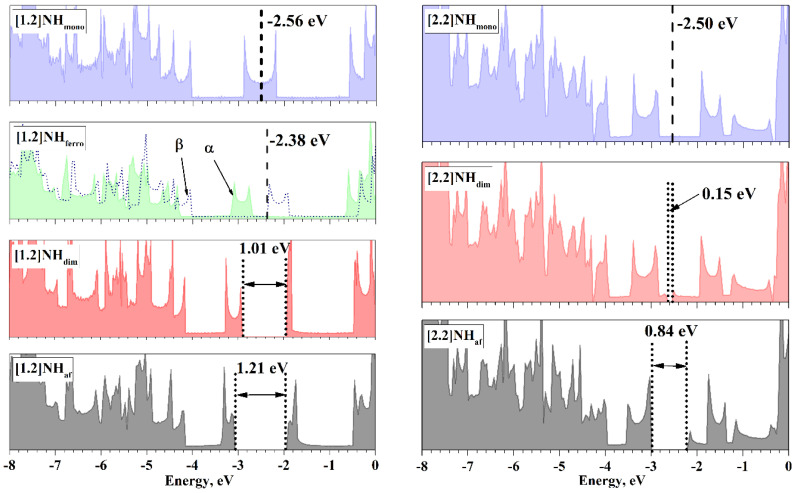
DOS of the NH at the point close to the energy minimum. For the [1.2]helicene, this is *L*41_6_ and *L*41_3_ in the case of the monomeric and dimeric states, respectively. For the [2.2]helicene, this is *L*56_25_ and *L*28_25_ in the case of the monomeric and dimeric states, respectively. Fermi energies are shown by dashed lines. The top of the valence band, TVB, and the bottom of the conduction band, BCB, are indicated by dotted lines. α and β indicate the corresponding spin DOS in the case of the [1.2]NH_ferro_.

Figure 4 shows the total densities of the electronic states (DOS) for different states of the considered structures (with a rational *Q*) closest to the energy global minima. The DOS of the states of the [2.2] helicene is expectedly more complicated than the DOS of states of the [1.2]helicene, but in all cases, Figure 4 shows the van Hove singularities that are typical for the Q1D systems.

The values of the Fermi energy of metallic states are close for both NHs, but there is a difference. The Fermi level of the [1.2]NH_mono_ is located in the middle of the area of high density in contrast to the Fermi level of the [2.2]NH_mono_. The ferromagnetic ordering shifts α-DOS towards lower energies, allowing the high-density region of the DOS to be completely filled with an excess of α-spin electrons and determines the existence of the [1.2]NH_ferro_ and the absence of the [2.2]NH_ferro_.

Both types of MIT open an electronic band gap (BG) near the Fermi level. In both NHs, the BG, opened due to the Peierls MIT, is narrower than that due to the Mott–Hubbard MIT. This becomes clear if we take into account the fact that the appearance of the BG corresponds to a decrease in the total energy, and the values of the BG can be a measure of this decrease.

The form of the DOS changes little under the MIT. These changes concern only the appearance of the BG. The changes in the rest of the DOS regions are also minimal, demonstrating that the electronic and atomic structures remain unchanged during the MIT.

The increase in *m*, i.e., the transition from the [1.2]helicene to the [2.2]helicene, expectedly reduces the BG of both types, confirming the results of [38]. The sharpest decrease occurs for the “dim” state, by more than 0.8 eV, making the [2.2]NH_dim_ state close to the highly symmetric [2.2]NH_mono_ state, as was already noted above. It can be assumed that with a further increase in *m*, for example, to *m* = 3, the states [3.2]NH_mono_ and [3.2]NH_dim_ will become degenerate, and the Peierls MIT will no longer take place.

The decrease in the BG of the “af” states with an increasing *m* from 1 to 2 is 0.37 eV, i.e., the “af” state is not going down as sharply as the “dim” state. A very rough estimate suggests that the MIT of this type disappears at *m* = 4 or 5, and such NHs will be in the “mono” state.

**Figure 5:**
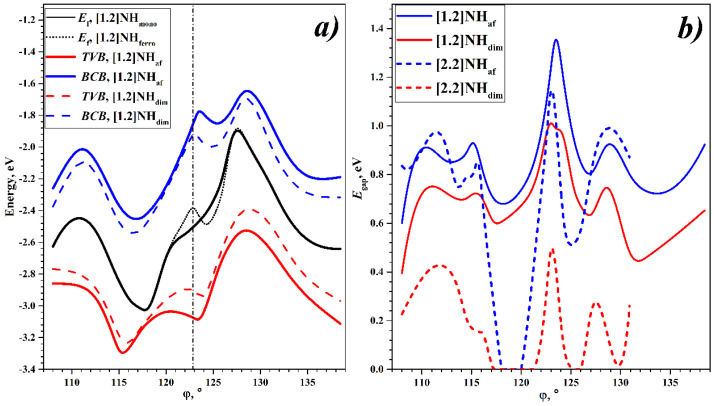
(**a**) Torsion dependence of *E_f_*, TVB, and BCB of [1.2]helicene’s states. (**b**) Torsion dependence on BG values, *E*_gap_, of the [1.2]helicenes and [2.2]helicenes.

The torsion deformation has a significant effect on NHs’ electronic properties. The change in the parameters of the electronic structure of the [1.2]helicene, in particular, and the positions of the Fermi energy, *E_f_*, the *top of the valence band*, TVB, and the *bottom of the conduction band*, BCB, during the torsion distortion are presented in Figure 5a. The Fermi energy of the “mono” state increases when φ increases from the minimum of the energy and decreases when φ decreases. The difference between the minimum and maximum values is about 1 eV. Upon reaching the maximum and minimum energy values, further torsion deformations give a reverse trend, and a final form of the *E_f_*(mono, φ) curve is shown in Figure 5a.

The torsion *E_f_*(ferro, φ) curve for the [1.2]NH_ferro_ differs from the curve for the [1.2]NH_mono_ in the region φ ~ 120–122.5°, i.e., the region of the existence of this state. An additional maximum and minimum appear on the *E_f_*(ferro, φ) curve, and the angle φ, corresponding to the new maximum, coincides with the φ value of the energy minimum.

Upon spontaneous symmetry breaking, the electronic states corresponding to the Fermi energy split into the conduction and valence bands. The energy dependences of the TVB and BCB on the rotational angle make it possible to estimate the effect of the torsion deformation on the MIT of both types. According to Figure 5a, the TVB and BCB change significantly with the torsion deformation. However, the general form of these torsion curves is similar to that of the *E_f_*(mono, φ) curve for the [1.2]NH_mono_ and differs only by a decrease and increase of about 0.2–0.3 eV of the TVB and BCB relative to the *E_f_* value, respectively.

Thus, the driving force of each MIT gives approximately the same influence on the electronic structure and depends little on the rotation angle. The TVB and BCB of the Peierls MIT are located inside the Mott–Hubbard MIT TVB and BCB in the entire considered angle region. This is consistent with the fact that the Peierls MIT energy is less than the Mott–Hubbard MIT energy over the entire angle region considered for the [1.2]helicene.

The dependence of the BG on the torsion distortion is shown in Figure 5b. For the [1.2]helicene, the states “dim” and “af” are semiconducting in the entire interval. The change in the BG for this NH is about 1 eV. The BG has maximum values in the region of the energy minimum, which coincides with the similar behavior of the BG of the first type of NHs [n,1] [44].

The band gap for the [2.2]helicene also exhibits a significant dependence on the torsion deformation. For the [2.2]NH_dim_ state, there are some rotation angle regions with a zero BG value. There are regions where the “dim” and the “mono” states are the same. Since the [2.2]NH_af_ state is more favorable than “dim”, this is not significant, but there is a region where the BG for the [2.2]NH_af_ is zero, and this region corresponds to the degeneracy of all three considered states, φ ~ (118–120°).

It should be noted that this region of the degeneracy of the [2.2]NH’s states is very close to the region of the global energy minimum. This gives an interesting possibility—starting from the antiferromagnetic global minimum with the value of *E*_gap_ = 0.8 eV, by means of a small torsion deformation, one can transform the [2.2]NH into a metallic diamagnetic system, having a degenerate state. In addition, further torsion deformation leads to a transition to an antiferromagnetic semiconductor with *E*_gap_ = 1.1 eV, which corresponds to a local minimum on the torsion energy curve for the [2.2]NH_af_ state. It should be emphasized that such a transition between the two minima with the manifestation of a metallic state is reversible since no bonds are broken in this transition—one coil simply slides around the other. This makes the [2.2]helicene a unique object for use in electro-magneto-mechanical nanodevices.

## 4. Conclusions

A quantum chemical study of the torsion energy curves of zig-zag-edged hexagonal NHs of the second type ([1.2] and [2.2], according to the classification of [38]) showed that these objects in their symmetric state (mono) are diamagnetic metals that are unstable with respect to the MIT. The consideration of two types of MIT (the Mott–Hubbard and Peierls) showed that for the considered NHs, the Mott–Hubbard MIT gives the greatest energy reduction in the process of spontaneous symmetry breaking; therefore, NHs of the second type are antiferromagnetic semiconductors in the regions of the energy minima.

The second type of NHs (with an even *n*), as well as the first type of NHs (with an odd *n*), are the structures that possess a helical periodicity only, without translational periodicity, and belong to the fifth family of the line symmetry groups in the global energy minimum regions. However, the torsion-induced symmetry breaking with the increase of the rotation angle, φ, leads to a transition to the first family of the line symmetry groups.

In addition to the antiferromagnetic state, a highly symmetric ferromagnetic state was found for the [1.2]helicene, the existence of which is possibly associated with the local effects of the rearrangement of the spin-polarized density. The energy of this state is higher than the energy of the antiferromagnetic state, but the existence of this state suggests that for highly complicated systems, in addition to the manifestation of the MIT, it is necessary to take into account the more subtle effects that can be considered by ab initio methods only.

For the [2.2]helicene, there is a region on the torsion energy curve where all the states are degenerate, and the [2.2]helicene in this region is a diamagnetic metal. This region is located between two energy minima corresponding to antiferromagnetic ordering. The transition from one to another leads to a reversible transformation through a diamagnetic metallic state that makes this NH very promising for use in electro-magneto-mechanical nanodevices.

## Figures and Tables

**Table 1:** Line symmetry group parameters used for calculations.

Monomeric States					Dimeric States				
φ, °	*Q*	*q*	*r*	*p*	φ’, °	*Q’*	*q’*	*r’*	*p’*
54.000	6.667	20	3	7	108.000	3.333	10	3	7
55.385	6.500	13	2	7	110.769	3.250	13	4	10
56.250	6.400	32	5	13	112.500	3.200	16	5	13
56.842	6.333	19	3	13	113.684	3.167	19	6	16
57.273	6.286	44	7	19	114.545	3.143	22	7	19
57.600	6.250	25	4	19	115.200	3.125	25	8	22
58.065	6.200	31	5	25	116.129	3.100	31	10	28
58.605	6.143	43	7	37	117.209	3.071	43	14	40
59.016	6.100	61	10	55	118.033	3.050	61	20	58
60.000	6.000	6	1	1	120.000	3.000	3	1	1
61.017	5.900	59	10	6	122.034	2.950	59	20	3
61.463	5.857	41	7	6	122.927	2.929	41	14	3
61.714	5.833	35	6	6	123.429	2.917	35	12	3
62.069	5.800	29	5	6	124.138	2.900	29	10	3
62.609	5.750	23	4	6	125.217	2.875	23	8	3
63.000	5.714	40	7	23	126.000	2.857	20	7	3
63.529	5.667	17	3	6	127.059	2.833	17	6	3
64.286	5.600	28	5	17	128.571	2.800	14	5	3

## Data Availability

Data will be made available on request.

## References

[1] Wang Y., Xu J., Wang Y., Chen H. (2013). Emerging chirality in nanoscience. Chem. Soc. Rev..

[2] Meng F., Morin S.A., Forticaux A., Jin S. (2013). Screw Dislocation Driven Growth of Nanomaterials. Acc. Chem. Res..

[3] Yang F., Wang M., Zhang D., Yang J., Zheng M., Li Y. (2020). Chirality Pure Carbon Nanotubes: Growth, Sorting, and Characterization. Chem. Rev..

[4] Wang P., Yang J., Sun G., Zhang X., Zhang H., Zheng Y., Xu S. (2018). Twist induced plasticity and failure mechanism of helical carbon nanotube fibers under different strain rates. Int. J. Plast..

[5] Yashima E., Maeda K., Iida H., Furusho Y., Nagai K. (2009). Helical Polymers: Synthesis, Structures, and Functions. Chem. Rev..

[6] Percec V., Rudick J.G., Peterca M., Wagner M., Obata M., Mitchell C.M., Cho W.-D., Balagurusamy V.S.K., Heiney P.A. (2005). Thermoreversible Cis-Cisoidal to Cis-Transoidal Isomerization of Helical Dendronized Polyphenylacetylenes. J. Am. Chem. Soc..

[7] Zhang S., Liu X., Li C., Li L., Song J., Shi J., Morton M., Rajca S., Rajca A., Wang H. (2016). Thiophene-Based Double Helices: Syntheses, X-ray Structures, and Chiroptical Properties. J. Am. Chem. Soc..

[8] Varni A.J., Fortney A., Baker M.A., Worch J.C., Qiu Y., Yaron D., Bernhard S., Noonan K.J.T., Kowalewski T. (2019). Photostable Helical Polyfurans. J. Am. Chem. Soc..

[9] Ikai T., Yoshida T., Shinohara K., Taniguchi T., Wada Y., Swager T.M. (2019). Triptycene-Based Ladder Polymers with One-Handed Helical Geometry. J. Am. Chem. Soc..

[10] Rickhaus M., Mayor M., Juríček M. (2016). Strain-induced helical chirality in polyaromatic systems. Chem. Soc. Rev..

[11] Shen Y., Chen C.-F. (2012). Helicenes: Synthesis and Applications. Chem. Rev..

[12] Daigle M., Morin J.-F. (2017). Helical conjugated ladder polymers: Tuning the conformation and properties through edge design. Macromolecules.

[13] Daigle M., Miao D., Lucotti A., Tommasini M., Morin J.-F. (2017). Helically coiled graphene nanoribbons. Angew. Chem. Int. Ed..

[14] Xiao X., Pedersen S.K., Aranda D., Yang J., Wiscons R.A., Pittelkow M., Steigerwald M.L., Santoro F., Schuster N.J., Nuckolls C. (2021). Chirality Amplified: Long, Discrete Helicene Nanoribbons. J. Am. Chem. Soc..

[15] Kiel G.R., Patel S.C., Smith P.W., Levine D.S., Tilley T.D. (2017). Expanded helicenes: A general synthetic strategy and remarkable supramolecular and solid-state behavior. J. Am. Chem. Soc..

[16] Zhan H., Zhang Y., Yang C., Zhang G., Gu Y. (2017). Graphene helicoid as novel nanospring. Carbon.

[17] Zhan H., Zhang G., Yang C., Gu Y. (2018). Breakdown of Hooke’s law at the nanoscale—2D material-based nanosprings. Nanoscale.

[18] Zhan H., Zhang G., Yang C., Gu Y. (2018). Graphene Helicoid: Distinct Properties Promote Application of Graphene Related Materials in Thermal Management. J. Phys. Chem. C.

[19] Sharifian A., Naeini V.F., Baniassadi M., Wu J., Baghani M. (2019). Role of Chemical Doping in Large Deformation Behavior of Spiral Carbon-Based Nanostructures: Unraveling Geometry-Dependent Chemical Doping Effects. J. Phys. Chem. C.

[20] Norouzi S., Fakhrabadi M.M.S. (2019). Nanomechanical properties of single- and double-layer graphene spirals: A molecular dynamics simulation. Appl. Phys. A.

[21] Sharifian A., Moshfegh A., Javadzadegan A., Afrouzi H.H., Baghani M., Baniassadi M. (2019). Hydrogenation-controlled mechanical properties in graphene helicoids: Exceptional distribution-dependent behavior. Phys. Chem. Chem. Phys..

[22] Liu R., Zhao J., Wang L., Wei N. (2020). Nonlinear vibrations of helical graphene resonators in the dynamic nano-indentation testing. Nanotechnology.

[23] Norouzi S., Kianfar A., Fakhrabadi M.M.S. (2020). Multiscale simulation study of anisotropic nanomechanical properties of graphene spirals and their polymer nanocomposites. Mech. Mater..

[24] Norouzi S., Fakhrabadi M.M.S. (2020). Anisotropic nature of thermal conductivity in graphene spirals revealed by molecular dynamics simulations. J. Phys. Chem. Solids.

[25] Zhu C., Ji J., Zhang Z., Dong S., Wei N., Zhao J. (2021). Huge stretchability and reversibility of helical graphenes using molecular dynamics simulations and simplified theoretical models. Mech. Mater..

[26] Zhu C., Liu M., Wei N., Zhao J. (2022). Molecular dynamics study on mechanical properties of helical graphenes/epoxy nanocomposites. Comput. Mater. Sci..

[27] Narjabadifam A., Abazadeh B., Fakhrabadi M.M.S. (2022). Graphyne nano-spirals under tension: Effects of base structures on superelasticity and fracture mechanisms. Mech. Mater..

[28] Li H., Afrouzi H.H., Zahra M.M.A., Bashar B.S., Fathdal F., Hadrawi S.K., Alizadeh A., Hekmatifar M., Al-Majdi K., Alhani I. (2023). A comprehensive investigation of thermal conductivity in of monolayer graphene, helical graphene with different percentages of hydrogen atom: A molecular dynamics approach. Colloids Surf. A.

[29] Korhonen T., Koskinen P. (2014). Electromechanics of graphene spirals. AIP Adv..

[30] Xu F., Yu H., Sadrzadeh A., Yakobson B.I. (2016). Riemann surfaces of carbon as graphene nanosolenoids. Nano Lett..

[31] Zhou Z., Yan L., Wang X.-M., Zhang D., Yan J.-Y. (2022). The sensitive energy band structure and the spiral current in helical graphenes. Results Phys..

[32] Treboux G., Lapstun P., Wu Z., Silverbrook K. (1999). Electronic conductance of helicenes. Chem. Phys. Lett..

[33] Tian Y.-H., Park G., Kertesz M. (2008). Electronic Structure of Helicenes, C_2_S Helicenes, and Thiaheterohelicenes. Chem. Mater..

[34] Avdoshenko S.M., Koskinen P., Sevinçli H., Popov A.A., Rocha C.G. (2013). Topological signatures in the electronic structure of graphene spirals. Sci. Rep..

[35] Zhang X.M., Zhao M.W. (2014). Strain-induced phase transition and electron spin-polarization in graphene spirals. Sci. Rep..

[36] Šesták P., Wu J., He J., Pokluda J., Zhang Z. (2015). Extraordinary deformation capacity of smallest carbohelicene springs. Phys. Chem. Chem. Phys..

[37] Xu X., Liu B., Zhao W., Jiang Y., Liu L., Li W., Zhang G., Tian W.Q. (2017). Mechanism of mechanically induced optoelectronic and spintronic phase transitions in 1D graphene spirals: Insight into the role of interlayer coupling. Nanoscale.

[38] Porsev V.V., Bandura A.V., Lukyanov S.I., Evarestov R.A. (2019). Expanded hexagonal nanohelicenes of zigzag morphology under elastic strain: A quantum chemical study. Carbon.

[39] He Y.-Y., Chen J., Zheng X.-L., Xu X., Li W.-Q., Yang L., Tian W.Q. (2019). Spiral Graphene Nanoribbons with Azulene Defects as Potential Nonlinear Optical Materials. ACS Appl. Nano Mater..

[40] Liu Z.-P., Guo Y.-D., Yan X.-H., Zeng H.-L., Mou X.-Y., Wang Z.-R., Wang J.-J. (2019). A metal-semiconductor transition in helical graphene nanoribbon. J. Appl. Phys..

[41] Liu Z.-P., Guo Y.-D., Zeng H.-L., Li J.-F., Jiang Y.-Y., Yan X.-H. (2020). Electrical control of spin polarization of transmission in pure-carbon systems of helical graphene nanoribbons. J. Appl. Phys..

[42] Thakur R., Ahluwalia P.K., Kumar A., Sharma R. (2021). Stability and electronic properties of bilayer graphene spirals. Phys. E.

[43] Mori K., Murase T., Fujita M. (2015). One-Step Synthesis of [16]Helicene. Angew. Chem. Int. Ed..

[44] Porsev V.V., Bandura A.V., Evarestov R.A. (2022). Ab initio modeling of helically periodic nanostructures using CRYSTAL17: A general algorithm first applied to nanohelicenes. Comput. Mater. Sci..

[45] Porsev V.V., Evarestov R.A. (2022). Quantum-mechanical calculation of the electronic band structure of helically periodic systems: Nanotubes and nanohelicenes. Phys. Solid State.

[46] Alexander E.Z. (1929). XVI. Systematik der eindimensionalen Baumgruppen. Z. Krist.-Cryst. Mater..

[47] Shubnikov A.V. (1940). Symmetry (The Laws of Symmetry and Their Application in Science, Technology, and Applied Art).

[48] Vujičić M., Božović I.B., Herbut F. (1977). Construction of symmetry groups of polymer molecules. J. Phys. A.

[49] Milošević I., Živanovic R., Damnjanović M. (1997). Symmetry classification of stereoregular polymers. Polymer.

[50] Damnjanović M., Milošević I. (2010). Line Groups in Physics. Theory and Applications to Nanotubes and Polymers.

[51] Damnjanović M., Milošević I. (2015). Full symmetry implementation in condensed matter and molecular physics—Modified group projector technique. Phys. Rep..

[52] Domnin A.V., Porsev V.V., Evarestov R.A. (2022). DFT modeling of electronic and mechanical properties of polytwistane using line symmetry group theory. Comput. Mater. Sci..

[53] Porsev V.V., Evarestov R.A. (2022). Ab initio modeling of helical polyacetylenes: Peierls and Mott-Hubbard metal–insulator transitions. Comput. Mater. Sci..

[54] Lazić N., Vuković N., Volonakis G., Milošević I., Logothetes, Damnjanović M. (2012). Natural torsion in chiral single-wall carbon nanotubes. J. Phys. Condens. Matter.

[55] Peierls R.E. (1955). Quantum Theory of Solids.

[56] Mott N.F. (1949). The Basis of the Electron Theory of Metals, with Special Reference to the Transition Metals. Proc. Phys. Soc. A.

[57] Damnjanović M., Vujičić M. (1982). Magnetic line groups. Phys. Rev. B.

[58] Dovesi R., Erba A., Orlando R., Zicovich-Wilson C.M., Civalleri B., Maschio L., Rérat M., Casassa S., Baima J., Salustro S. (2018). Quantum-mechanical condensed matter simulations with CRYSTAL. WIREs, Comput. Mol. Sci..

[59] Dovesi R., Pascale F., Civalleri B., Doll K., Harrison N.M., Bush I., D’Arco P., Noël Y., Rérat M., Carbonnière P. (2020). The CRYSTAL code, 1976–2020 and beyond, a long story. J. Chem. Phys..

[60] Ferrari A.M., Civalleri B., Dovesi R. (2010). Ab initio periodic study of the conformational behavior of glycine helical homopeptides. J. Comput. Chem..

[61] Perdew J.P., Ernzerhof M., Burke K. (1996). Rationale for mixing exact exchange with density functional approximations. J. Chem. Phys..

[62] Peintinger M.F., Oliveira D.V., Bredow T. (2013). Consistent Gaussian basis sets of triple-zeta valence with polarization quality for solid-state calculations. J. Comput. Chem..

[63] Monkhorst H.J., Pack J.D. (1976). Special points for Brillouin-zone integrations. Phys. Rev. B.

